# Association Between Perceived Stress and Thyroid Function in Female Healthcare Workers

**DOI:** 10.1192/j.eurpsy.2026.11534

**Published:** 2026-07-31

**Authors:** S. Chtiba, M. Bouhoula, S. Mrad, O. Salah, I. Kacem, A. Aloui, M. Makhloufi, A. Chouchane, H. Kalboussi, O. El Maalel, S. Chatti, B. Charfeddine, M. Maoua

**Affiliations:** 1 University of Sousse, Faculty of Medicine of Sousse; 2 Department of Biochemistry; 3 Department of Occupational Medicine, Farhat Hached University Hospital; 4 Department of Occupational Medicine, Sahloul University Hospital, Sousse, Tunisia

## Abstract

**Introduction:**

Healthcare workers are regularly exposed to high levels of occupational stress, which may lead to physiological and endocrine alterations. Thyroid function, known to be particularly sensitive to stress-induced hormonal variations, could be affected, especially among women, a population more prone to fatigue and hormonal imbalances.

**Objectives:**

To evaluate the relationship between perceived stress levels and thyroid function among female healthcare professionals.

**Methods:**

This cross-sectional study was conducted at the Department of Occupational Medicine and Occupational Pathologies of Farhat Hached University Hospital, Sousse, over a one-year period from February 1, 2024, to February 28, 2025.

Female healthcare professionals aged 25 to 49 years, with at least one year of professional experience, were randomly recruited after providing written informed consent.

Exclusion criteria included personal or family history of thyroid disorders or psychiatric illnesses, as well as the use of medications likely to affect thyroid function within the four months preceding the study.

Data were collected using a predesigned questionnaire covering sociodemographic and occupational characteristics, in addition to anthropometric parameters (weight, height, body mass index [BMI]). Perceived stress was assessed using the Cohen and Williamson Perceived Stress Scale (PSS-10). Serum concentrations of thyroid-stimulating hormone (TSH) and free thyroxine (fT4) were measured by electrochemiluminescence immunoassay.

Reference ranges were defined as follows: 0.25–4.5 mIU/L for TSH and 12–22 pmol/L for fT4.

**Results:**

A total of 91 female healthcare professionals were included in the study.

The mean age was 37.36 ± 5.26 years, ranging from 26 to 49 years.

Overall, 94.5% of participants had normal thyroid function (TSH < 4.5 mIU/L with normal fT4), while the remaining presented with subclinical hypothyroidism (TSH > 4.5 mIU/L with normal fT4).

Regarding stress levels, 7.7% had a low PSS score, 74.7% had a moderate score, and 17.6% had a high perceived stress score. No significant differences were observed between women with normal thyroid function and those with subclinical hypothyroidism.

A positive correlation was found between PSS scores and TSH levels (correlation coefficient = 0.058), although this association was not statistically significant.

**Conclusions:**

The findings of this study suggest a trend toward a positive correlation between perceived stress scores and serum TSH concentrations, although the association did not reach statistical significance. Further studies with larger sample sizes are warranted to better assess the impact of stress on thyroid function, particularly among female healthcare professionals.

**Disclosure of Interest:**

None Declared

